# Incidence of Chronic Kidney Disease and Its Risk Factors, Results of Over 10 Year Follow Up in an Iranian Cohort

**DOI:** 10.1371/journal.pone.0045304

**Published:** 2012-09-27

**Authors:** Maryam Tohidi, Mitra Hasheminia, Reza Mohebi, Davood Khalili, Farhad Hosseinpanah, Babak Yazdani, Amir Ahmad Nasiri, Fereidoun Azizi, Farzad Hadaegh

**Affiliations:** 1 Prevention of Metabolic Disorders Research Center, Research Institute for Endocrine Sciences, Shahid Beheshti University of Medical Sciences, Tehran, Iran; 2 Obesity Research Center, Research Institute for Endocrine Sciences, Shahid Beheshti University of Medical Sciences, Tehran, Iran; 3 Department of Nephrology, Masih Daneshvari Hospital, Shahid Beheshti University of Medical Sciences, Tehran, Iran; 4 Endocrine Research Center, Research Institute for Endocrine Sciences, Shahid Beheshti University of Medical Sciences, Tehran, Iran; University of Louisville, United States of America

## Abstract

To examine, the predictors of incident chronic kidney disease (CKD) in a community-based cohort of Middle East population, during a mean follow-up of 9.9 years. In a sample of 3313 non-CKD Iranian adults ≥20 years the estimated glomerular filtration rate (eGFR) was calculated at baseline and at three year intervals during three consecutive phases. The eGFR <60 mL/min/1.73 m2 was defined as CKD. Multivariate Logistic regression analysis was used to determine the independent variables associated with incident CKD. The incidence density rates of CKD were 285.3 and 132.6 per 10,000 person-year, among women and men, respectively. Female gender per se was associated with higher risk of CKD, compared with males. Among women, age, eGFR, known diabetes, being single or divorced/widowed, hypertension (marginally significant) and current smoking were independent risk factors for CKD; however the intermediate degree of education and family history of diabetes decreased the risk by 40% (P<0.05). Among male subjects, independent predictors of developing CKD included aging and hypertension (with significantly higher risk than in women, P for interaction<0.05), eGFR, new diagnosed diabetes, high normal blood pressure; abdominal obesity decreased the risk of CKD about 30% which was marginally significant. In the Iranian population,>2% of individuals develops CKD each year. Our findings confirmed that sex- specific risk predictors should be considered in primary prevention for incident CKD.

## Introduction

There is a rising prevalence and incidence of chronic kidney failure (CKD), with poor outcomes and high cost in the world [1]. In fact, the numbers of the patients with end stage renal disease (ESRD) on renal replacement therapy, in the United States, Japan and most European countries have increased by 9%, 7% and 4%per year, respectively [2], [3]. It is estimated that by 2030, over 70% of patients with ESRD will be inhabitants of developing countries [4],probably related to the fast rising trend of obesity and diabetes in these countries [5]. To the best of our knowledge, all previous studies regarding the risk factors for incident chronic kidney disease (CKD) in community based cohort study have been conducted in United States, Europe and Asia[6]–[8]. Among these studies, however, only a few reported important gender differences in the predictors of CKD [7], [9].

The prevalence of CKD among the Iranian population is known to be high [10], [11]; these studies reporting the same related factors including female gender for prevalent CKD in the whole population. However no report about the long term incidence of CKD has yet been published from Middle Eastern countries with high prevalence of risk factors for CKD including diabetes and hypertension [5]. We aimed to determine the incidence of CKD and its associated risk factors in a large community based cohort study of Tehranain, selected from among the participants of the Tehran Lipid and Glucose Study (TLGS). Furthermore we investigated whether gender is an important modifier for risk factors of incident CKD among this population.

## Materials and Methods

### Study Population

**Figure 1:**
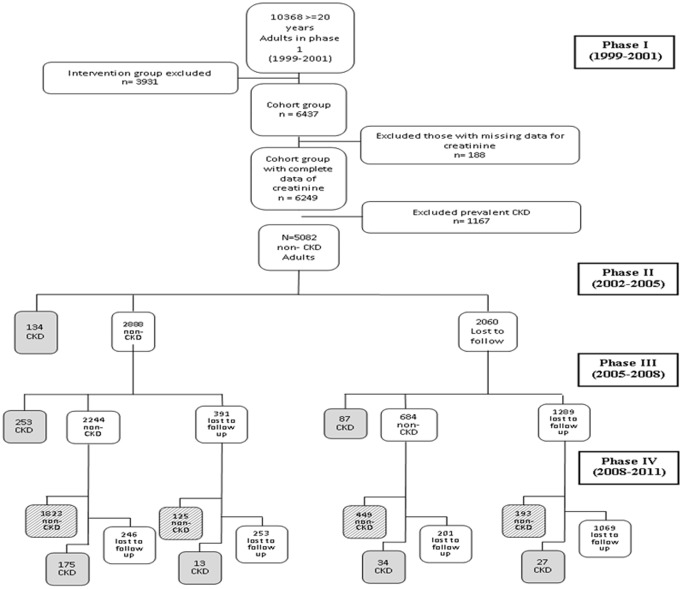
Follow up status of the TLGS participants after the baseline examination. CKD; chronic kidney disease.

In brief, the TLGS is a large scale, long term, community-based prospective study performed on a representative sample of residents of district No. 13 of Tehran, capital of Iran [12]. Age and sex distributions of the population in the district were representative of the overall population of Tehran at the time of the baseline examination. The TLGS, which has two major components: a cross-sectional prevalence study of non-communicable disease (1999 to 2001) and associated risk factors, implemented between March 1999 and December 2001, and a prospective follow-up study. A total of 27, 340 residents aged ≥3 years were invited by telephone call, of which 15, 010 residents participated in first examination phase. After this cross-sectional prevalence study of NCD risk factors, subjects entered into a cohort and a prospective interventional study, the latter to be educated for implementation of life style changes. The cohort group consisted of 6437 subjects, aged ≥20 years. After exclusion of subjects with prevalent CKD stages3 to 5(CKDs3-5) at baseline (n = 1167) and those with missing data regarding creatinine level (n = 188), there were 5082 non- CKDs3-5 subjects in the cohort group, who entered phase 2 TLGS (Figure 1). Those who developed CKDs3-5 in the follow-up examinations (phases 2 or 3 or 4) and those who completed the fellow-up at phase 4 examination were included in the current study (n = 3313). The main reasons for lack of attendance at follow-up examinations despite repeated calls were either personal or migration. The proposal of this study was approved by the research council of The Research Institute for Endocrine Sciences of Shahid Beheshti University of Medical Sciences and informed written consent was obtained from each subject.

Subjects were interviewed privately, by trained interviewers, using pretested questionnaires. Initially, information on demographics, education, smoking status, medical and drug history was collected. Anthropometric measures including weight, height, waist circumference (WC) was measured according to a standard protocol [12]. Body mass index (BMI) was calculated as weight in kg divided by height in m^2^. Systolic and diastolic blood pressures were measured twice in a seated position in the right arm and the mean value was considered as the subject's blood pressure. A blood sample was taken after 12–14 h overnight fasting and was centrifuged within 30–45 min of collection. All blood analyses were performed at the TLGS research laboratory on the day of blood collection. For oral glucose tolerance test, 82.5 g glucose monohydrate solution (equivalent to 75 g anhydrous glucose) was administered orally to subjects and a blood sample was taken 2 hours later.

Fasting and 2-hours plasma glucose (FPG and 2-hPG respectively) were measured by enzymatic colorimetric glucose oxidase method; both inter-and intra-assay coefficient of variations (CV) were less than 2.2%. Total cholesterol (TC) and triglycerides (TG) were assayed using the enzymatic calorimetric method with cholesterol esterase- cholesterol oxidase and glycerol phosphate oxidase, respectively. For both total and HDL-Cholesterol, intra-and inter-assay CVs were 0.5 and 2% respectively. Intra and inter-assay CVs were 0.6 and 1.6% for TG respectively.

Serum creatinine (cr) levels were assayed by kinetic colorimetric Jaffe. The sensitivity of the assay was 0.2 mg/dL (range, 18–1330 µmol/L (0.2–15 mg/dL). Reference intervals according to manufacturer’s recommendation were 53–97 µmol/L (0.6–1.1 mg/dL) and 80–115 µmol/L (0.9–1.3 mg/dL) in women and men respectively. Both intra-assay and inter-assay CVs were less than 3.1% in both baseline and follow-up phases. All biochemical assays were performed using commercial kits (Pars Azmoon Inc., Tehran, Iran) by a Selectra 2 auto analyzer (Vital Scientific, Spankeren, The Netherlands). Assay performance was monitored after every 25 tests using lyophilized serum controls in normal and pathologic ranges and all samples were analyzed when internal quality control met the standard acceptable criteria. [10], [12].

### Definition of Variables and Outcomes

According to the Kidney Disease Outcome Quality Initiative guidelines, chronic kidney disease is defined as either kidney damage or Glomerular Filtration Rate (GFR) <60 mL/min/1.73 m2 for >3 months [13]. For this study GFR was estimated using the abbreviated prediction equation, provided by the Modification of Diet in Renal Disease (MDRD) study as follows:

Abbreviated MDRD study equation:
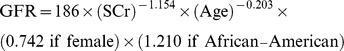



In this equation, eGFR (estimated GFR) is expressed as mL/min per 1.73 m2 and serum creatinine (Scr) is expressed as mg/dL [14].

Incident CKD was considered an eGFR below than 60 mL/min/1.73 m2 occurring at any time during the follow-up period. This corresponds to stage 3 to stage 5 CKD based on the Kidney Disease Outcomes and Quality Initiative guidelines.

Body mass index was categorized in to 3 groups of <25 kg/m2 (reference), 25 to <30 kg/m2 (overweight), and ≥30 kg/m2 (obese). Abnormal waist circumference was set at ≥90 cm for both genders, as defined for Iranian populations [15]. According to the 2007 European guidelines for the management of arterial hypertension, we categorized subjects into four groups : 1.The optimal blood pressure group,those with systolic blood pressure (SBP) lower than 120 mmHg and diastolic blood pressure (DBP) lower than 80mmHg (reference); 2. The normal BP group, those with BP between 120 and 129 mmHg or DBP between 80 and 84 mmHg as normal BP group; 3.The high normal BP group, those with SBP between 130 and 139 mmHg or DBP between 85 and 89 mmHg; 4.The hypertensive group, those with SBP higher than 140 mmHg or DBP higher than 90 mmHg or taking antihypertensive medications. If systolic and diastolic pressures fell into different categories, participants were assigned to the higher category [16].

Dyslipidemia was defined as serum triglycerides of ≥2.26 Mmol/l or cholesterol of ≥6.19 Mmol/l and included subjects taking lipid lowering medications [17]. Smoking was defined in 3 groups; 1. Participants who smoked cigarettes daily or occasionally as well as those who used water pipe or pipe as current smokers; 2. participants who smoked in the past; and 3. Those who never smoked (reference). Education was categorized into 3 groups: 1. illiterate/primary school; 2. cycle/diploma and 3. Higher than diploma (reference). Marital status categorized as single, married (reference), widowed/divorced. Positive family history of diabetes was defined as having at least one parent or sibling with diabetes. History of cardiovascular disease (CVD) was defined as previous ischemic heart disease and/or cerebrovascular accidents. Participants were classified as known diabetes mellitus (KDM) if they reported having medication treated diabetes. According to the 1997 diagnostic criteria of the American Diabetes Association (ADA) [18], people without known diabetes were categorized as follows: Normal glucose tolerance (NGT)(reference), fasting plasma glucose (FPG) <5.6 and 2h-PG <7.8 mmol/l; Newly diagnosed diabetes mellitus (NDM), FPG ≥7.0 or 2h-PG≥11.1 mmol/l; Impaired fasting glucose (IFG), FPG ≥5.6 and <7.0 mmol/l and impaired glucose tolerance(IGT), 2h-PG≥7.8 and <11.1 mmol/l (in the current study we pooled IFG and IGT cases as a single group of IFG/IGT). Age groups included; 20 to ≤34years (reference), 35 to ≤49 years, 50 to ≤64 years and ≥65 years in both genders. eGFR was categorized as;<75 (reference); ≥75 and <90; ≥90 mL/min/1.73 m2.

### Statistical Analyses

Mean (standard deviation: SD) values for continuous and frequencies (%) for categorical variables of the baseline characteristics are given for participants with and without incident CKDs3-5. Since FPG, 2h-PG and TG had skewed distribution they are shown as median (interquartile range). Comparison of baseline characteristics between participants with and without incident CKDs3-5 was done by student’s t-test for continuous variables, chi-square test for categorical variables and Mann-Whitney test for skewed variables.

To reduce selection bias [19],propensity scores,the estimated probability that a participant would have followed in the study, were computed using maximum likelihood logistic regression analysis in both genders and in the whole population as well. For this reason, the entire baseline measures including FPG, 2hPG, TG, TC, SBP, DBP, BMI, marital status, education level, history of CVD, age, eGFR, waist circumference, drug consumption for diabetes, lipid and hypertension, family history of diabetes and smoking status were included in a logistic model as exposures with participation in the follow-up as the outcome; the probability of participation in follow-up was then estimated for every participants.

The association of different categorical risk factors with incident CKDs3-5 was assessed by calculating multivariate adjusted odds ratios (ORs) with 95% CI using binary logistic regression analysis. For risk factors with more than 2 categories the first category was considered as the reference group. Each candidate predictor (age categories, eGFR categories, hypertension categories, diabetes groups, dyslipidemia, smoking status, history of CVD, family history of diabetes, educational levels, marital status,general and abdominal obesity) with a p-value less than 0.2 in the initial univariable analysis was included in the multivariable analysis. The probability of participation in follow-up was used as a propensity score, which added to the logistic models as a covariate. This probability was associated with incident CKDs3-5 in the multivariate model among women (p<0.001). However, we entered the propensity score in models running among men and whole population as well. The selection bias, therefore, probably did not affect our estimations.

In multivariate analysis, the effect modification of gender on the relation between other covariates and CKDs3-5 outcome were tested by entering the interaction terms (covariate × gender) in the model; there were significant effect modification of gender on all age groups (all Ps <0.05), new diagnosed diabetes (P<0.001), high normal blood pressure (P = 0.05) and hypertension (P = 0.001). Hence, we stratified our analysis by gender. Also, for our findings to be comparable to other studies, we showed our data analyses in the whole population as well. All P-values were two-tailed. P-value ≤0.05 being considered statistically significant. Statistical analyses were performed using SPSS program (SPSS Inc., Chicago, IL, USA; Version 15).

## Results

As shown in Table 1, women participants were older (38.33 vs. 36.24 years) and had lower eGFR (72.99 vs. 74.56 mL/min/1.73 m2), but higher TC (5.34 vs. 5.21 Mmol/l), and higher waist circumference (85.90 vs.84.77 cm) compared with nonparticipants. Men participants had lower history of CVD (3.7 vs. 6.4%) and reported lower consumption of anti-diabetic medications (1.7 vs.4.1%), but had higher TC (5.28 vs. 5.12 Mmol/l) and higher TGs (1.77 vs. 1.59 Mmol/l) than nonparticipants. Additionally, in both genders there was a significant difference in marital status between participants vs. nonparticipants.

**Table 1:** Comparison of baseline characteristics between followed up vs. non-followed up in the TLGS cohort*.

	Men				Women		
	Non-followed up (N = 837)	Followed up (N = 1454)	P-V		Non- Followed up (N = 932)	Followed up(N = 1859)	P-V
Age(years)	41.46(15.78)	41.35(13.43)	.86		36.24(12.95)	38.33(11.99)	<.001
eGFR(ml/min/1.73m^2^)	76.26(10.11)	75.69(10.04)		.19	74.56(9.86)	72.99(9.13)	<.001
FPG(mmol)/l	5.05(4.72–5.50)	5.05(4.72–5.44)		.24	4.88(4.55–5.27)	4.88(4.61–5.27)	.68
2-hPG (mmol)/l	5.49(4.44–6.73)	5.55(4.49–6.77)		.79	5.83(4.91–7.05)	5.88(4.94–7.05)	.85
TC(mmol)/l	5.12(1.13)	5.28(1.13)		.001	5.21(1.20)	5.34(1.22)	.008
TG(mmol)/l	1.59(1.08–2.35)	1.77(1.23–2.52)		<.001	1.39(0.96–2.07)	1.40(0.97–2.14)	.25
SBP(mmHg)	119.68(19.43)	119.06(16.69)		.44	115.06(116.83)	115.46(16.98)	.56
DBP(mmHg)	77.15(11.40)	77.75(10.67)		.22	76.65(10.04)	76.81(9.91)	.68
Waist(cm)	87.16(11.80)	87.97(11.21)		.10	84.77(13.56)	85.90(12.25)	.04
BMI(kg/m^2)^	25.34(4.25)	25.61(4.06)		.13	26.82(5.48)	27.21(4.83)	.07
DM Drug (%)	4.1	1.7		.001	2.9	2.5	.53
Lipid Drug (%)	1.6	1.1		.44	2.7	3.0	.81
HTN Drug (%)	4.3	2.9		.09	4.5	5.4	.31
Marital status (%)				.008			<.001
Married	75.4	80.8			73.9	84.1	
Divorced/Widowed	0.6	0.6			6.1	5.4	
Single	24.0	18.6			20.0	10.5	
HCVD (%)	6.4	3.7		.005	2.0	2.1	.89
FHDM (%)	24.6	26.1		.45	26.8	28.2	.47
Smoking (%)				.15			.09
Never	54.1	58.1			92.2	94.3	
Past	13.6	13.3			2.3	1.8	
Current	32.4	28.7			5.5	3.8	
Education Level (%)				.31			.21
Higher than diploma	19.4	19.0			11.3	9.8	
Diploma/Cycle	56.2	59.2			59.7	58.5	
Illiterate/Primary School	24.4	21.8			29.0	31.8	

TLGS; Tehran lipid and glucose study,CKD; chronic kidney disease, P-V; p-value, eGFR; estimated glomerular filtration rate, FPG; fasting plasma glucose, 2-hPG; 2-hours plasma glucose,TC; total cholesterol, TG; triglyceride, SBP; systolic blood pressure, DBP; diastolic blood pressure, BMI; body mass index, DM; diabetes mellitus, HTN; hypertension, HCVD, history of cardiovascular disease,FHDM; family history of diabetes mellitus.

Table 2 shows the baseline characteristics of the 3313 participants with and without incident CKD. In both genders, the participants who developed incident CKDs3-5 were older, had lower eGFR but higher SBP and DBP, lipid levels, FPG, 2-hPG,BMI and waist circumference and were more likely to be smokers (only in women) compared with participants free of CKDs3-5 at the end of follow-up (P<0.05 for all of these measures).

**Table 2:** Baseline characteristics of subjects who did and did not develop incident CKD stage 3-5 after 10 years of follow-up.

	Men			Women		
	Non-CKD (N = 1248)	CKD(N = 206)	P-V	Non-CKD (N = 1342)	CKD(N = 517)	P-V
Age(years)	39.28(12.56)	53.87(11.64)	<.001	35.52(10.67)	45.63(12.17)	<.001
eGFR(ml/min/1.73m^2^)	76.99(9.83)	67.78(7.34)	<.001	74.97(9.28)	67.84(6.31)	<.001
FPG(mmol)/l	4.99(4.66–5.38)	5.33(4.88–5.97)	<.001	4.88(4.55–5.22)	4.99(4.66–5.49)	<.001
2-hPG (mmol)/l	5.49(4.38–6.6)	5.99(4.83–8.63)	<.001	5.83(4.88–6.94)	6.05(5.05–7.55)	.008
TC(mmol)/l	5.24(1.13)	5.54(1.06)	<.001	5.2(1.15)	5.71(1.35)	<.001
TG(mmol)/l	1.75(1.2–2.51)	1.91(1.4–2.56)	.034	1.31(0.92–2.02)	1.68(1.12–2.39)	<.001
SBP(mmHg)	117.3(15.1)	129.5(21.56)	<.001	113.45(15.30)	120.7(19.79)	<.001
DBP(mmHg)	77.0(10.23)	82.31(12.07)	<.001	75.92(9.5)	79.14(10.57)	<.001
Waist(cm)	87.5(11.18)	90.6(11.04)	<.001	84.55(12.07)	89.37(12.03)	<.001
BMI(kg/m^2)^	25.52(4.08)	26.18(3.88)	.035	26.74(4.8)	28.4(4.6)	<.001
DM Drug (%)	1.0	6.0	<.001	1.3	5.7	<.001
Lipid Drug (%)	1.1	1.5	.48	1.7	6.1	<.001
HTN Drug (%)	1.8	9.5	<.001	3.3	10.8	<.001
Marital status (%)			<.001			<.001
Married	78.4	95.6		83.7	85.1	
Divorced/Widowed	0.5%	1.5		3.2	11.0	
Single	21.2	2.9		13.1	3.9	
HCVD (%)	2.9	9.0	<.001	1.4	3.9	.002
FHDM (%)	26.2	25.4	.86	29.1	26.0	.20
Smoking (%)			.69			.008
Never	58.1	58.0		95.4	91.7	
Past	13.0	15.0		1.6	2.4	
Current	28.9	27.0		3.0	5.9	
Education Level (%)			<.001			<.001
Higher than diploma	19.8	14.1		10.1	8.9	
Diploma/Cycle	61.1	47.6		64.8	42.1	
Illiterate/Primary School	19.1	38.3		25.1	49.0	

CKD; chronic kidney disease, P-V; p-value, eGFR; estimated glomerular filtration rate, FPG; fasting plasma glucose,2-hPG; 2-hours plasma glucose,TC; total cholesterol, TG; triglyceride, SBP, systolic blood pressure, DBP, diastolic blood pressure, BMI; body mass index, DM, diabetes mellitus, HTN; hypertension, HCVD, history of cardiovascular disease, FHDM; family history of diabetes mellitus.

Overall, 723 new cases CKDs3-5 were identified after a mean follow-up 9.9 years (minimum = 7.2, maximum = 12.3 years) resulting in a crude cumulative incidence of 21.8% (95%CI: 20.42–23.23). The corresponding cumulative incidence among women and men were 27.8% (517/1859), (95% CI: 25.77–29.85), and 14.2% (206/1454), (95% CI: 12.38–15.96), respectively. The incidence density rate of CKDs3-5 among the whole population was 214.82 (95% CI 199.72–231.07) per 10,000 person/years; and corresponding incidence rates among women and men were 285.3 (95% CI 261.8–311.0) and 132.6 (95% CI 115.7–152.0), respectively.

Table 3 shows the adjusted ORs of CKDs3-5 at 10 years of follow-up associated with baseline risk factors. Baseline -adjusted predictors of developing CKDs3-5 among women included age (subjects with aged ≥50 years had more than a doubling of OR), eGFR, known diabetes [OR,6.20(2.68–14.36)], being single or divorced/widowed compared with married status increased the OR by 3 fold, hypertension with a marginal OR of about 40%, current smoker [5.74(2.71–12.15)];however, the presence of intermediate degree of education and the presence of family history of diabetes significantly decreased the risk of incident CKDs3-5 by 40%.

**Table 3:** The predictors for developing CKD stage 3–5 during 10-year follow-up by gender in the TLGS cohort.

	Men		Women	
	Odds ratio (CI)	p-value	Odds ratio (CI)	p-value
Age(years)				
20–34	Reference		Reference	
35–49	3.36 (1.41–8.02)	0.01	1.17(0.83–1.63)	0.37
50–64	10.85(4.44–26.55)	<0.001	2.54(1.54–4.20)	<0.001
≥65	13.47(4.72–38.47)	<0.001	2.76(1.15–6.63)	0.02
eGFR(ml/min/1.73m^2^)				
<75	Reference		Reference	
≥75 & <90	0.27(0.16–0.43)	<0.001	0.39(0.27–0.56)	<0.001
≥90	0.07(0.01–0.52)	0.01	0.25(0.07–0.86)	0.03
Diabetes Group				
None	Reference		Reference	
IGT/IFG	1.04(0.66–1.64)	0.88	0.98(0.70–1.36)	0.89
New diagnosedDM	2.48(1.37–4.51)	0.003	0.98(0.58–1.66)	0.94
Known DM	2.17 (0.68–6.90)	0.19	6.20 (2.68–14.36)	<0.001
Marital status				
Married	Reference		Reference	
Divorced/Widowed	1.23(0.16–9.70)	0.85	2.94(1.63–5.29)	<0.001
Single	0.89(0.30–2.65)	0.84	3.08(1.24–7.64)	0.02
HCVD				
No	Reference		Reference	
Yes	0.88(0.40–1.94)	0.75	1.44(0.67–3.10)	0.35
Education Level				
Higher than diploma	Reference		Reference	
Diploma/Cycle	1.13(0.68–1.88)	0.65	0.62(0.41–0.94)	0.03
Illiterate/Primary School	0.95(0.53–1.68)	0.85	0.80(0.50–1.28)	0.34
Hypertension				
Optimal	Reference		Reference	
Normal	1.11(0.65–1.88)	0.70	0.94(0.68–1.29)	0.69
High normal	1.74(0.99–3.04)	0.05	1.23(0.81–1.87)	0.32
Hypertension	2.20(1.38–3.52)	0.001	1.40(0.96–2.06)	0.08
Dyslipidemia				
No	Reference		Reference	
Yes	1.38(0.93–2.03)	0.11	0.79(0.60–1.05)	0.11
Abdominal Obesity				
No	Reference		Reference	
Yes	0.70(0.48–1.04)	0.08	0.98(0.71–1.35)	0.91
BMI(kg/m2 )				
<25	Not Applicable*		Reference	
≥25 & <30			1.26(0.91–1.74)	0.17
≥30			1.39(0.91–2.10)	0.12
Smoking				
Never	Not Applicable*		Reference	
Past			1.67(0.66–4.22)	0.28
Current			5.74(2.71–12.15)	<0.001
FHDM				
No	Not Applicable*		Reference	
Yes			0.63(0.48–0.84)	0.002

Odds ratios were obtained by multivariate logistic regression analysis. Dyslipidemia; TG of ≥2.26 Mmol/l or TC of ≥6.19 Mmol/l or subjects taking lipid lowering medications. Abdominal Obesity; waist circumference≥90 in both genders, TLGS; Tehran lipid and Glucose Study, eGFR; estimated glomerular filtration rate,IGT; Impaired glucose test, IFG; Impaired fast glucose, DM, diabetes, HCVD, history of cardiovascular disease, FHDM; family history of diabetes mellitus, BMI; Body mass index.* Had p-values >0.2 in the initial univariable analysis and were not included in the multivariable analysis.

Among male subjects, adjusted predictors of developing CKD included aging,hypertension,high normal BP (which had significantly higher risk than in women), eGFR, new diagnosed diabetes [2.48(1.37–4.51];interestingly abdominal obesity decreased the risk of CKDs3-5 about 30% which was marginally significant.

Finally, in the whole population, in which the female gender per se was associated with more than tripling risk of CKD compared with men(3.17,95% CI 2.44–4.12), age, eGFR, known diabetes,current smoking and hypertension were independent predictors.(Table 4).

**Table 4:** The predictors for developing CKD stage 3–5 during 10-year follow-up period in the whole population.

	Total	
	Odds ratio(CI)	p-value
Sex		
Male	Reference	
Female	3.17(2.44–4.12)	<0.001
Age(years)		
20–34	Reference	
35–49	1.61(1.21–2.14)	0.001
50–64	5.12(3.59–7.28)	<0.001
≥65	7.67(4.44–13.24)	<0.001
eGFR(ml/min/1.73m^2^)		
<75	Reference	
≥75 &<90	0.29(0.22–0.38)	<0.001
≥90	0.11(0.04–0.30)	<0.001
Diabetes Group		
None	Reference	
IGT/IFG	0.95(0.73–1.24)	0.72
New diagnosed DM	1.21(0.81–1.80)	0.36
Known DM	2.89(1.41–5.89)	0.004
Marital status		
Married	Reference	
Divorced/Widowed	1.28(0.80–2.05)	0.30
Single	0.68(0.38–1.22)	0.20
HCVD		
No	Reference	
Yes	1.34(0.76–2.35)	0.31
Education Level		
Higher than diploma	Reference	
Diploma/Cycle	0.81(0.59–1.12)	0.20
Illiterate/Primary School	0.81(0.57–1.16)	0.26
Hypertension		
Optimal	Reference	
Normal	0.89(0.68–1.16)	0.38
High normal	1.25(0.90–1.72)	0.19
Hypertension	1.43(1.08–1.91)	0.01
Dyslipidemia		
No	Reference	
Yes	0.95(0.75–1.20)	0.65
Abdominal Obesity		
No	Reference	
Yes	0.85(0.65–1.11)	0.23
BMI(kg/m^2^ )		
<25	Reference	
> = 25 &<30	1.12(0.86–1.47)	0.39
> = 30	1.1(0.77–1.57)	0.60
Smoking		
Never	Reference	
Past	0.81(0.52–1.27)	0.36
Current	1.45(0.99–2.13)	0.06

Odds ratios were obtained by multivariate logistic regression analysis.TLGS; Tehran lipid and Glucose Study, eGFR; estimated glomerular filtration rate, IGT; Impaired glucose test, IFG; Impaired fast glucose, DM, diabetes, HTN; hypertension, HCVD, history of cardiovascular disease, BMI; Body mass index. Dyslipidemia; TG of ≥2.26 Mmol/l or TC of ≥6.19 Mmol/l or subjects taking lipid lowering medications. Abdominal Obesity; waist circumference≥90 in both genders.

## Discussion

According to the results of the present study, in the Iranian population, aged 20 years and over, more than 2% of individuals developed CKDs3-5 each year, during 10 years follow-up. Among whole Iranian population, age, female gender, eGFR, known diabetes, current smoking and hypertension were found to be significant independent predictors for incident CKDs3-5. Additionally in sex stratified analysis among women, being single or divorced/widowed relative to being married were positive predictors, while intermediate degree of education and family history of diabetes were both negative predictors for incident CKDs3-5. Among men, newly diagnosed diabetes and high normal blood pressure was independent predictors.

In a combined cohort of 2 community-based studies, the Atherosclerosis Risk in Communities Study (ARIC) and the Cardiovascular Health Study (CHS), in a population aged 45 years or older, during a follow-up period of 9 years, 9.9% (ARIC) and 16.9% (CHS )developed incident CKDS3-S5,respectively [20]. Among Framingham Offspring participants, (mean age 43 years), free of stage 3 CKD at baseline, 7.9% developed stage 3 CKD over an 18.5 year follow-up [21]. In Japan, during the 10 years follow-up among 123 764 adults aged 40 years in a community-based cohort 19 411 subjects (male: 4257, female: 15 154) developed CKD stage III or higher [3] . Despite the lower mean age of study population, the reported incidence in the current study was higher than similar population based studies in the US, Europe and Japan [6]–[8], [20], [21]. One possible explanation for this observation is the high prevalence and incidence of type 2 diabetes and hypertension among Iranian population [22]–[24].

In a previous study of a Tehranian population, factors independently associated with CKD were age, female gender, BMI, high waist circumference, hypertension and dyslipidemia [25]. In another study from Northern Iran, based on multivariate analysis, age, female sex and self-reported hypertension were significantly associated with prevalent CKD [11]. In line with previous cross sectional studies conducted among Iranian population, we found that female gender per se increased the risk of incident CKDs3-5 by more than 3fold. In National Health and Nutrition Examination Survey, females had 30% higher risk than males for prevalent CKD [26]. Importantly, as reported by Bash L et al, the association between sex and incident CKD differed in direction and extent, based on different definitions of CKD [27].

Among risk factors analyzed in our study, aging was found as a significant predictor for CKD in both genders, as reported in other studies [6]–[8]: however the effect of aging on kidney function was more prominent in our male population. In line with our findings, Halbesma et al [9] based on a new method of slope-based analysis (rather than threshold analysis) showed that the decline in the mean of eGFR slope over time was significantly higher in males than in females. However, among a general Japanese population GFR decreased at a similar rate in both genders in all age groups [8].

We found known diabetes in the whole population and new diagnosed diabetes among men as significant risk factors for incident CKDs3-5. The lack of association between known diabetes and incident CKD among men might be related to lack of power, considering the wide confidence interval [2.144(0.667–6.888)]. Given the high prevalence and incidence of Type 2 diabetes and very low prevalence of Type 1 diabetes among an Iranian population, our results primarily reflect Type 2 diabetes [22].

In the current study we did not find any independent risk for the general or central adiposity measure in prediction of incident CKD. In line with our findings, in the Framingham Heart Study, the significant association of general obesity with stage 3 CKD disappeared after considering known CVD risk factors [21]. The loss of association between obesity measures and incident CKDs3-5 after risk factor adjustment suggest that the relation between obesity and CKDS3-S5 may be attributed to the presence of diabetes or hypertension. In 26 year follow-up in Eastern Finland, the independent effect of obesity on ESRD risk seemed to appear only just before the end of study [28]; hence, the lack of independent risk of obesity in our study might be related to the medium duration of follow-up. Interestingly, among men higher WC was associated with a lower risk for incident CKDs3-5 which was marginally significant [0.703(0.476–1.040), P = 0.078]; a similar finding among the male population of Hoorn study was shown,applying change in renal function as the incident CKD rather than using a predefined threshold [9].It could be speculated that lower risk of incident CKD among TLGS population with abdominal adiposity might be attributable to healthier life because of increased awareness among cohort population however, we recently [29] demonstrated that there is increasing trend in the prevalence of general and abdominal obesity among TLGS participants during phase 2 and 3 in both genders.

According to the national survey, approximately 25% Iranians, aged 25–64 years had hypertension, and among of whom, 25% were taking antihypertensive medications, of these treated subjects, only 24% had BP values <140/90 mmHg [24]. In the current study, we found hypertension as a strong predictor for incident CKD, as observed in cross-sectional and population based cohort studies [10], [11]. In the current study BP≥140/90 mmHg or using antihypertensive medications were associated with incident CKDs3-5 however; an association marginally significant among women. Furthermore, only among men high normal BP was significantly associated with incident CKDs3-5. Similar to our findings, in the Halbesma et al study, a higher systolic blood pressure among males was associated with more renal function decline than in women [9].

Smoking arises as an important preventable renal risk factor based on studies highlighting a strong association of smoking and renal damage in men and women.Furthermore, some studies show favorable effects of smoking cessation on kidney function [30]. In the current study, despite the lower prevalence of smoking among women compared with men, the current female smokers showed over a 5 fold risk for incident CKDs3-5.

Surprisingly, we found that among women, despite significant risk of known diabetes for incident CKD, the presence of family history of diabetes resulted in more than 35% lower risk for incident CKDs3-5 in multivariate analysis; findings which are speculated to be attributable to better dietary patterns among women with positive family history of diabetes. Furthermore, considering education as the socio-economic criteria of the study population we found Iranain women with intermediate degree of education (i.e. diploma or cycle) showed lower risk for CKDs3-5 compared to those with higher education.

In our study population we showed the lower mean eGFR in non-CKD 3-5 study population at the baseline, similar to the eGFR reported in two other populations based cohort studies conducted in Japan [3] and Netherland [9] for determining incident CKD which was 80 (SD: 14 ml/min/1.73 m2), that falls into CKD 2 category per Kidney Disease Outcomes and Quality Initiative guidelines [14]. So there is a concern if the different levels of eGFR might modify the effect of other predictors of incident CKD3-5. To examine this concern we tested the interaction (as the effect modification measurement) between eGFR and each covariate in multivariate model; eGFR was not effect modifier for other predictors (p>0.05) except age in men (p = 0.01) and family history of diabetes in women (p = 0.04). The effect of these predictors was a little higher in higher levels of eGFR, since the study population had a low level of eGFR at baseline we might somehow underestimate the effect of these risk factors only (Data available on request).

Limitations of our study include first, we measured the baseline characteristics of the participants only once, and hence misclassification of potential risk factors such as blood pressure categories might attenuate our estimates. We based our diagnosis of CKD on a single estimate of eGFR, which we acknowledge tends to overestimate the incidence of kidney disease. Estimated GFR measurements exhibit a high degree of intra-individual variability and ideally require second measurements to accurately represent kidney function. The use of successive eGFR measurements, had they been available, would likely have reduced the incidence of CKDs3-4 but would not attenuated the association of the predictor variables with the outcome. Furthermore, most studies of CKD, epidemiologic and interventional, use single serum creatinine measurements. Second, we did not calibrate our serum creatinine measurements to the Cleveland Clinic, where the Modification of Diet in Renal Disease (MDRD) eGFR equation was derived; nor did we validate the MDRD eGFR equation in a local population, and this could also cause an overestimation in the incidence of CKDs3-5. Third, the differences between respondents (i.e. 15, 010 individuals) and non-respondents (i.e. 12,240 individuals ) in the TLGS cohort at baseline(1999–2001) [31] showed that the respondents had higher self-reported history of diabetes, hypertension,dyslipidemia and lower history of smoking than nonrespondents. Furthermore, Among women, comparison between followed and non- followed population showed that the former had higher age and lower mean eGFR than the later at baseline; the issue that was translated to significant and positive PS only among women(data not shown).Hence, it seems that participants of TLGS cohort, in particular women, had higher risk for incident CKD than general population so we overestimated the incidence of CKD. Finally, there are uncertainties about generalizability of our results to other geographic regions of the country.

As strengths, this study includes the continued follow-up of the TLGS population and the actual measurement of CVD risk factors and laboratory parameters, rather than self-reported data. Furthermore, our cohort was not designed selectively for CKD events, which precludes possible referral or selection bias. Finally, we defined CKDS3-5 as outcome is our study, since the DMARD estimation formula, originated among persons with a baseline GFR <60ml/min/1.73 m2, is most precise for individuals at this stage of kidney function [32].

To conclude, during 10 years follow-up, the cumulative incidence of CKDs3-5 among women and men were 27.8%and 14.2%, respectively. Age, hypertension and diabetes were found to be independent predictors of CKDs3-5 in both genders, with greater risks among men than women. Additionally, in males, high normal BP and, among females, current smoking and being single or divorced/widowed was a significant risk factor. Among females, family history of diabetes and intermediate degree of education were independently associated with a better renal function outcome, whereas in males abdominal obesity per se (marginally significant) had this association. Our findings confirm that sex- specific risk predictors should be considered in primary prevention for incident CKD among an Iranian population.
